# Gender income differences among general practitioners with compulsory services in early career stage in underdeveloped areas: evidence from a prospective cohort study in China

**DOI:** 10.1186/s12960-024-00930-z

**Published:** 2024-06-27

**Authors:** Haoqing Tang, Mingyue Li, Huixian Zheng, Xiaotian Zhang, Xiaoyun Liu

**Affiliations:** 1 Department of Health Policy and Management, School of Public Health, Peking University, Beijing, China; 2 China Center for Health Development Studies, Peking University, 38 Xueyuan Road, Haidian District, Beijing, 100191 People’s Republic of China; 3 School of Health Policy and Management, Nanjing Medical University, Nanjing, China

**Keywords:** Gender difference, General practitioner, Income, Underdeveloped areas, China

## Abstract

**Background:**

Gender equality and the gender income gap in medicine are long-standing global problems. Although gender-related differences have been widely studied in developed countries, they remain unclear in underdeveloped regions. In 2010, China initiated a national compulsory service program (CSP) to train qualified general practitioners in rural and remote areas. This study aimed to evaluate gender income differences for early career CSP and non-CSP (NCSP) graduates in underdeveloped areas.

**Methods:**

A cohort study was conducted with 3620 CSP and NCSP graduates from four medical universities in Central and Western China. Baseline surveys and six follow-up surveys were conducted between 2015 and 2022. Incomes, including monthly mean income and proportion of performance-based income, were measured as the key outcome variables. Multivariate linear regression models were used to identify the gender income gap.

**Results:**

NCSP graduates had higher average monthly incomes than CSP graduates. In the seventh year after graduation, the average monthly income for NCSP graduates was 7859 CNY while was 5379 CNY for CSP graduates. After controlling for demographic characteristics, the gender monthly income gap for CSP graduates was expanded from the fourth year (3.0%) to the sixth year (5.9%) after graduation, and that for NCSP graduates was expanded from the fifth year (11.9%) to the seventh year (16.3%) after graduation. Regarding performance-based income, it was 58.9% for NCSP graduates and 45.8% for CSP graduates in the seventh year after graduation. After controlling for performance-based income proportion, the gender income gap was reduced from 5.9 to 4.0% in the sixth year after graduation for CSP graduates, and from 16.3 to 14.4% for NCSP graduates in the seventh year after graduation.

**Conclusion:**

An extensive and ever-increasing gender income gap exists among young doctors in the early stages of their careers in underdeveloped areas of China. The high proportion of performance-based income among men is one of the main explanations for the observed difference. A more explicit compensation system must be established to enhance support for female health workers.

**Supplementary Information:**

The online version contains supplementary material available at 10.1186/s12960-024-00930-z.

## Highlights

- A seven-year cohort study examined the gender income gap for practitioners in early career stage.
- An increasing gender income gap exists for doctors in underdeveloped areas in China.
- The income gap was smaller for those trained by China’s compulsory services program.
- Higher performance-based income for men is the main explanation for the income gap.

**Supplementary Information:**

The online version contains supplementary material available at 10.1186/s12960-024-00930-z.

## Introduction

Women account for approximately 75% of the global health workforce [1]. In China, medicine has recently undergone a strong shift toward feminization; in 2021, 73.0% of health workers were women, compared to 63.0% in 2002 [2]. Gender inequality and the gender career income gap in medicine are long-standing problems globally [3–6]. Many studies across developed countries have shown that male doctors are more likely to be promoted and earn more than female doctors. A previous review showed that the total medical gender pay gap in England was 24.4% for hospital doctors, 33.5% for general practitioners, and 21.4% for clinical academics in 2020 [3]. A 2012 longitudinal study in Australia found that female general practitioners (GPs) earned 24.9% less than male GPs [7]. Another cross-sectional study conducted in Iran in 2019 found that male GPs earned 35.3% more than female GPs [8]. Women often earn less than men due to factors such as the “carer effect”, which refers to the stronger sense of responsibility women feel for child-rearing after having children [9], labor market discrimination [10], and differences in the types of services [11]. There are significant disparities in the choice of specialties between men and women, resulting in horizontal gender segregation in career development paths. For example, 60% of female GPs frequently perform gynecologic follow-ups compared to 24% of male GPs, while male GPs more often perform electrocardiograms, minor surgeries, and traumatology [11]. Early career gender disparities can set long-term trends in workforce distribution, leading to gender imbalances among healthcare professionals across various specialties [12]. This can potentially create shortages in critical areas and impact overall healthcare delivery. Additionally, diversity in healthcare providers can improve the quality of care by ensuring a wider range of perspectives and approaches. Gender disparities in early career stages can reduce this diversity, potentially affecting patient outcomes [13]. Therefore, gender differences in the early stage of career development may have a more significant impact on the development of healthcare manpower and the provision of healthcare services.

Low income and low financial incentive have been widely regarded as key reasons for health workforce shortage in China’s primary health care system [14, 15]. In 2010, China began a national compulsory services program (CSP) for medical students. The program trains GPs in rural areas in China’s Central and Western regions, with the goal of improving the capacity of rural primary health care (PHC) services and increasing health accessibility and equity. CSP students are not required to pay for tuition or accommodations during their five-year undergraduate studies, and they may receive monthly allowances. In return, they must commit to working as GPs at appointed primary health care systems for six years after graduation. In contrast, most non-CSP (NCSP) students choose to work as specialists in county and above level hospitals. Seven years have passed since the first wave of students graduated from the CSPs. Income is a crucial factor influencing whether GPs remain in primary health care systems [16]. Furthermore, the earnings disparity between genders has repercussions for the retention of GPs, with a notable influence on the labor supply of female GPs [17]. This effect becomes particularly pronounced as the proportion of female GPs in the workforce increases, yet it has received insufficient attention in both policy practice and academic research.

The compensation structure for doctors in China typically comprises a fixed base salary, performance-based earnings, and various allowances [18]. Moreover, an annual year-end bonus is provided, with bonuses often representing a significant proportion of the overall salary [19]. Currently, in China, there is widespread overdependence on performance incentives to motivate healthcare professionals. Zhang and Liu discovered that years of practice, educational background, technical title, management position, and specialty all influenced doctors' salaries [19]. However, there is a notable evidence gap regarding the effects of performance incentives on income disparities among GPs from a gendered perspective.

To date, most studies on the CSP have focused on health workforce attraction, recruitment, and retention in rural and remote areas [20–22]. However, there has been minimal research on gender differences and the determinants of doctors’ income in underserved areas, despite financial incentive playing an important role in influencing attraction, retention, and development. As gender also has implications for the availability and acceptability of health workers in rural communities [23–25], more research on gender differences among doctors at different levels is required. Previous studies on gender differences were mostly from developed countries and regions [13, 26, 27]. Little attention has been paid to the gender difference among doctors working in middle- and low-level economic regions, where women's social status is usually lower [28–30].

Therefore, we sought to evaluate gender income differences in a new population of young medical graduates in underdeveloped areas who were early in their careers. The "underdeveloped areas" focused on in this study primarily refer to rural areas of middle- and low-level economic regions in China. The study period ranged from the year 2015 to 2022. Our goal was to gain an up-to-date understanding of the exact level and change in the income gap between CSP and NCSP graduates and to evaluate whether the gender differences previously observed among health workers would be apparent in this younger and more recently hired cohort. In addition, we examined how performance-based income determines gender differences. This study provides evidence for other countries seeking to increase access for healthcare workers in underdeveloped areas.

## Methods

### Study design and data collection

#### Study design

Data for this study were obtained from the Cohort Study of Medical Graduates with Compulsory Services in Rural Areas, a prospective cohort study of Chinese medical graduates. The study was established in 2015 and looks at medical education, residency training, employment, and career development to help China's rural and remote regions grow their health workforce. The Institutional Review Board (IRB) of Peking University Health Science Center provided ethics approval for this study (IRB00001052-15027). All participants provided informed consent.

#### Baseline data collection

The study was launched in 2015 and has established five sub-cohorts in five years of medical graduates. A nonrandom purposive sample of four medical universities which undertook CSP were chosen from western and central China, representing underdeveloped areas (middle- and low-level economic regions) of China. The three provinces, Guangxi, Jiangxi and Qinghai, are all below the national average level in GDP and household consumption (Table S1). The survey included 3620 medical graduates from Qinghai University (Qinghai Province, Northwest China), Guangxi Medical University (Guangxi Zhuang Autonomous Region, Southwest China), Jiujiang University, and Gannan Medical University (Jiangxi Province, Central China).

After five years of undergraduate study, the first group of CSP-trained medical professionals graduated in 2015. This group formed the first sub-cohort, and we gathered baseline data from the four medical schools. The CSP classes were matched 1:1 with NCSP classes from the same year. Sub-cohorts were also created from the 2016, 2017, 2018, and 2019 classes. Participants completed a paper questionnaire at baseline before completing their undergraduate studies. Data on demographics, employment, postgraduate studies, residency training, and employment-related information were collected from both types of graduates.

The key predictor in this study, gender, was identified as female or male (based on the dichotomous response options available in the baseline questionnaire).

#### Follow-up data collection

In the baseline survey, we established WeChat groups encompassing all participants within each school and sub-cohort. WeChat is a widely used instant messaging application in China that facilitates communication between investigators and participants. After a baseline survey, annual online follow-up surveys were conducted. By 2022, we had successfully completed six follow-up surveys for the 2015 graduates. Links to online self-administered questionnaires were disseminated annually via email, the WeChat groups, and mobile text messages. Follow-up information was collected in 2016, 2017, 2018, 2020, 2021, and 2022. Due to logistical reasons, the 2019 follow-up survey was not conducted for the 2015 to 2018 graduates.

The outcome variables in this study were average monthly income (in Chinese Yuan [CNY]) and the proportion of performance-based income. Average monthly income was assessed using the question, "What is the monthly income for this job?" Performance-based income was gauged using the question, "In this context, how much of the income is based on performance?" All variables related to income were adjusted using the Consumer Price Index (CPI) in 2015.

A number of labor market variables widely postulated to influence income were available in the follow-up questionnaire, including demographic characteristics (marital status and education level), workload (outpatient volume and inpatient volume), and work-related characteristics (current workplace, job performance, whether participants passed China National Medical Licensing Examinations, whether they finished standardized training for resident physicians, and whether they received a title or job promotion).

Unemployed individuals and those who did not work in underdeveloped areas were excluded. The final sample comprised 2039 CSP graduates and 1571 NCSP graduates.

### Statistical analysis

We categorized our cohorts based on number of years since graduation. This was done to investigate income disparity patterns between males and females in the early stages of their professional development and to observe how this discrepancy evolved with each additional year since graduation. Descriptive analysis was used to identify the characteristics of the study sample and distribution of medical graduates’ income (average monthly income and proportion of performance-based income) between men and women. A t-test was used to compare the differences between men and women and CSP and NCSP graduates for each successive year after graduation. Following a log-transformation of the income variable to account for data distribution skewness, we employed multiple linear regressions to assess the gender income gap for each year since graduation, the independent associations among gender and other sample characteristics, and work-related characteristics associated with differences in occupational earnings.

All statistical analyses were conducted using STATA (version 17.0; Stata Corp., College Station, TX, USA). P values less than 0.05 were considered statistically significant.

## Results

Table 1 presents the basic characteristics of the study sample. A total of 3620 medical graduates were included in the baseline survey from 2015 to 2019, including 2041 CSP graduates, accounting for 56.4%. However, 10 individuals were excluded due to missing gender information. After exclusion, the baseline sample size was 3610. A similar number of men and women participated (50.6% male and 49.3% female).

**Table 1:** Basic characteristics of the study sample [*n* (%)]

	Basic characteristics of the study sample		
	Male (*n* = 1829)	Female (*n* = 1781)	Overall (*n* = 3610)
*Yeas of graduation*			
2015	323 (17.7%)	297 (16.7%)	620 (17.2%)
2016	383 (20.9%)	356 (20.0%)	739 (20.5%)
2017	434 (23.7%)	373 (20.9%)	807 (22.4%)
2018	351 (19.2%)	392 (22.0%)	743 (20.6%)
2019	338 (18.5%)	363 (20.4%)	701 (19.4%)
*School*			
Qinghai	473 (25.9%)	668 (37.5%)	1141 (31.6%)
Guangxi	445 (24.3%)	486 (27.3%)	931 (25.8%)
Jiujiang	291 (15.9%)	189 (10.6%)	480 (13.3%)
Gannan	620 (33.9%)	438 (24.6%)	1058 (29.3%)
*Type*			
CSP	1066 (58.3%)	973 (54.6%)	2039 (56.5%)
NCSP	763 (41.7%)	808 (45.4%)	1571 (43.5%)

Table 2 shows the characteristics of the study sample for CSP and NCSP graduates in the latest follow-up survey in 2022. Most CSP graduates (74.1%) worked in CHC and THC, whereas most NCSP graduates (90.7%) worked in public hospitals at the county level and above. Most NCSP graduates (69.4%) had received postgraduate qualifications, while only 4.6% of CSP had postgraduate qualifications. Most medical graduates (97.4%) had passed the China National Medical Licensing Examinations, and all graduates had completed standardized training for resident physicians. The NCSP graduates had a higher workload than the CSP graduates, both in terms of the number of outpatients (25.8 vs. 18.9) and inpatients (32.1 vs. 24.8). Although the graduates had at most seven years of experience, 83.0% of CSP graduates and 68.5% of NCSP had been given title promotions. Of the CSP graduates, 16.0% had received job promotions, whereas only 2.5% NCSP graduates had been promoted.

**Table 2:** Characteristics of the study sample in the latest follow-up survey 2022 [*n*(%)]

	CSP (*n* = 2039)		NCSP (*n* = 1571)	
	Male	Female	Male	Female
Total	1066	973	763	808
Married	316 (58.7%)	231 (48.5%)	133 (43.5%)	125 (39.9%)
Current workplace				
Public hospitals at county level and above	131 (26.7%)	91 (21.5%)	226 (91.9%)	222 (89.5%)
CHC & THC	350 (71.4%)	325 (76.7%)	3 (1.2%)	6 (2.4%)
Other	9 (1.8%)	8 (1.9%)	17 (6.9%)	20 (8.1%)
Education				
Postgraduate qualifications	12 (2.2%)	33 (6.9%)	215 (70.2%)	215 (68.6%)
Passed China National Medical Licensing Examinations	866 (96.7%)	815 (98.5%)	482 (97.0%)	511 (97.3%)
Finished standardized Training for residents Physicians	720 (100.0%)	619 (100.0%)	290 (100.0%)	307 (100.0%)
High job performance (≥ 72)	161 (29.9%)	104 (21.8%)	89 (29.1%)	79 (25.2%)
Workload (mean (SD))				
Outpatient numbers	18.9 (23.9)	19.0 (27.4)	24.8 (32.8)	26.7 (42.7)
Inpatient numbers	27.0 (63.0)	22.6 (50.0)	36.2 (56.2)	27.9 (55.2)
With title of attending doctor	267 (49.6%)	183 (38.4%)	35 (11.4%)	31 (10.2%)
With job promotion	84 (15.6%)	54 (11.3%)	5 (1.6%)	8 (2.6%)

The total score of the job performance scale was 12 items × 7 points = 84; *CHC* community health center, *THC* township health center

Table 3 describes the differences in average monthly income between men and women for each successive year after graduation for both CSP and NCSP graduates. For both men and women, NCSP graduates had a higher average monthly income than CSP graduates in each year since graduation, and the difference between CSP and NCSP graduates widened over the seven years (from 379 CNY in year one to 2479 CNY in year seven). The average monthly income of CSP graduates in their seventh year after graduation was 5379 CNY per month, which was a significantly lower change than that of NCSP graduates (7859 CNY per month). Throughout the seven years, no significant gender gap existed in the average monthly incomes of CSP graduates. However, statistically significant gender differences were found among NCSP graduates who had been practicing for five to seven years, and the gender gap in the seventh years was 1058.7 CNY.

**Table 3:** Difference in average monthly income between men and women for each successive year after graduation for both CSP and NCSP graduates

Graduation duration	Average month income															
	CSP (*n* = 2039)							NCSP (*n* = 1571)							Difference^b^ (CNY)	*P* value
	Male		Female		Difference^a^ (CNY)	*P* value	Total mean (CNY)	Male		Female		Difference^a^ (CNY)	*P* value	Total mean (CNY)		
	*n*	Mean (CNY)	*n*	Mean (CNY)				*n*	Mean (CNY)	*n*	Mean (CNY)					
1	688	2588	617	2885	− 297	0.000	2728	180	3165	204	3034	131	0.339	3107	− 379	< 0.001
2	638	3203	602	3419	− 216	0.012	3308	162	3962	160	3866	96	0.630	3914	− 606	< 0.001
3	659	3411	578	3583	− 171	0.046	3489	216	4967	232	4827	139	0.491	4897	− 1408	< 0.001
4	426	3641	374	3799	− 157	0.082	3717	164	6138	155	5752	385	0.160	5950	− 2233	< 0.001
5	381	3972	311	4121	− 149	0.112	4038	162	6764	189	6178	586	0.034	6448	− 2409	< 0.001
6	184	4787	143	4938	− 151	0.329	4857	107	7871	130	7003	867	0.020	7394	− 2537	< 0.001
7	84	5293	48	5531	− 237	0.268	5379	71	8377	68	7318	1058	0.023	7859	− 2479	< 0.001

(1) Difference^a^ = male–female; Difference^b^ = CSP-NCSP. (2) Average month income was adjusted using the Consumer Price Index (CPI). (3) CNY, Chinese yuan renminbi

Table 4 presents the differences in the proportions of performance-based income between men and women in each year since graduation for CSP and NCSP graduates. From the second year after graduation, CSP graduates had a significantly higher proportion of performance-based income than NCSP graduates. The proportion of performance-based income for those that had graduated seven years ago was 48.7% for CSP graduates and 58.9% for NCSP graduates. Among CSP graduates, men in their fourth to seventh years after graduation received a significantly higher proportion of performance-based income than women. The gender gap increased from 2.3 (fourth year) to 4.6% (seventh year). Among NCSP graduates, men in their second to seventh years after graduation received a significantly higher proportion of performance-based income than women. The gender gap increased from 1.6 (second year) to 8.5% (seventh year).

**Table 4:** Differences in the proportions of performance-based income between men and women in each year since graduation for CSP and NCSP graduates

Graduation duration	The proportion of performance-based income															
	CSP (*n* = 2039)							NCSP (*n* = 1571)							Difference^b^	*P* value
	Male		Female		Difference^a^	*P* value	Total	Male		Female		Difference^a^	*P* value	Total		
	*n*	Mean	*n*	Mean				*n*	Mean	*n*	Mean					
1	–	–	–	–	–	–	–	–	–	–	–	–	–	–	–	–
2	638	22.4%	602	22.4%	0.0%	0.888	22.4%	162	42.4%	160	40.8%	1.6%	0.003	41.6%	− 19.2%	< 0.001
3	659	28.4%	578	28.0%	0.4%	0.395	28.2%	216	50.4%	232	45.5%	4.9%	0.000	47.8%	− 19.6%	< 0.001
4	426	26.9%	374	24.6%	2.3%	0.005	25.8%	164	54.3%	155	49.2%	5.1%	0.000	51.9%	− 26.1%	< 0.001
5	381	36.0%	311	33.3%	2.7%	0.001	34.8%	162	57.7%	189	51.2%	6.5%	0.000	54.2%	− 19.4%	< 0.001
6	185	44.9%	143	41.0%	3.9%	0.002	43.2%	107	59.5%	130	53.3%	6.2%	0.006	56.1%	− 12.9%	< 0.001
7	84	50.4%	48	45.8%	4.6%	0.014	48.7%	71	63.0%	68	54.5%	8.5%	0.011	58.9%	− 10.2%	< 0.001

(1) Difference^a^ = male–female; Difference^b^ = CSP-NCSP. (2) Performance-based income was adjusted using the Consumer Price Index (CPI). (3) CNY, Chinese yuan renminbi

The results of the multivariate regression analysis of the average monthly incomes of the CSP and NCSP graduates are presented in Table 5. After controlling for the covariates of school, years since graduation for each sub-cohort, marital status, current workplace, and whether they had received a title or job promotion, we found a statistically significant difference between genders in average monthly income (Model 1). Among CSP graduates, men had higher average monthly income than women, and the gender income gap widened between the fourth and sixth years after graduation. Women earned 3.0% less than men in the fourth year after graduation, and the gap increased to 5.9% in the sixth year after graduation. The insignificance of the gender gap in the seventh year may be related to the relatively small sample size (only 2015 graduates). For NCSP graduates, men also had higher average monthly income than women, and the gender income gap widened between the fifth and seventh years after graduation. Women earned 11.9% less than men in the fifth year after graduation, and the gap increased to 16.3% in the seventh year after graduation. The gender income gap was significantly greater among NCSP graduates (16.3%) compared to CSP graduates (3.2%) in the seventh year after graduation.

**Table 5:** Multivariate regression analysis of the average monthly incomes of the CSP and NCSP graduates

	Graduation duration	Model	Sex (ref: Male)	The proportion of performance-based income	Marital status (ref: Not married)	Workplace (ref: Public hospitals at county level and above)		Title promotion (ref: Without title)	Job promotion (ref: Without administrative positions)
			Female		Married	CHC & THC	Other	With title	With administrative positions
CSP (*n* = 2039)	1	Model 1	0.019	–	0.017	− 0.091***	0.068	–	–
		Model 2	–	–	–	–	–	–	–
	2	Model 1	0.028	–	− 0.034	− 0.032	0.188	0	− 0.072
		Model 2	0.029	0.219	− 0.034	− 0.032	0.158	0.002	− 0.069
	3	Model 1	− 0.01	–	− 0.003	− 0.115***	0.18	− 0.004	− 0.003
		Model 2	− 0.008	0.394***	− 0.004	− 0.119***	0.199	− 0.002	0.002
	4	Model 1	− 0.030*	–	0.007	− 0.155***	0.096	0.006	0.024
		Model 2	− 0.021	0.494***	0.005	− 0.139***	0.159	− 0.007	0.016
	5	Model 1	− 0.038*	–	0.009	− 0.101***	− 0.067	0.023	0.068**
		Model 2	− 0.023	0.661***	0.018	− 0.093***	− 0.084	0.026	0.066**
	6	Model 1	− 0.059*	–	− 0.035	− 0.142***	0.046	0.097	0.099**
		Model 2	− 0.04	0.584***	− 0.039	− 0.129***	0.074	0.124*	0.096**
	7	Model 1	− 0.032	–	− 0.057	− 0.100**	− 0.02	0.114	0.027
		Model 2	− 0.02	0.383*	− 0.053	− 0.089*	0.002	0.11	0.027
NCSP (*n* = 1571)	1	Model 1	− 0.011	–	0.055	− 0.023	0.164*	–	–
		Model 2	–	–	–	–	–	–	–
	2	Model 1	− 0.231	–	0.23	–	–	0.315	–
		Model 2	− 0.317	− 0.789	0.248	–	–	0.35	–
	3	Model 1	0.063	–	0.059	0.256	0.106	0.091	− 0.145
		Model 2	0.1	0.633*	0.066	0.294	0.137	0.079	− 0.154
	4	Model 1	− 0.035	–	0.103*	0.131	0.178	0.144**	− 0.154
		Model 2	− 0.015	0.438*	0.093	0.116	0.179*	0.137*	− 0.164
	5	Model 1	− 0.119**		− 0.075	− 0.091	0.264**	0.162***	0.004
		Model 2	− 0.100*	0.288	− 0.074	− 0.079	0.266**	0.168***	0.000
	6	Model 1	− 0.103*	–	0.011	− 0.133	− 0.118	0.271***	0.192
		Model 2	− 0.099*	0.082	0.011	− 0.123	− 0.116	0.271***	0.19
	7	Model 1	− 0.163**	–	0.079	0.091	0.021	0.173	0.395**
		Model 2	− 0.144*	0.176	0.069	0.129	0.025	0.146	0.353*

(1) In the first year, there is no data of title promotion, job promotion and performance-based income. (2) *** *P* < 0.001, ** *P* < 0.01, * *P* < 0.05; *β* coefficient and confidence interval were reported. (3) The schools and years of graduation for each sub-cohort were controlled for in all regressions

When we included the proportion of performance-based income in Model 2, the gender income gap was reduced. In the seventh year after graduation, for CSP graduates, the income gap decreased by 1.2% points compared to the gap in Model 1. Meanwhile, for NCSP graduates, the income gap also decreased by 1.9% points in the seventh year after graduation compared to the gap in Model 1. This finding provides further evidence that the proportion of performance-based income may be a reason for the gender income gap.

## Discussion

Gender income inequity continues to be pervasive in medicine [5, 12, 31, 32]. To our knowledge, this is the first study to use a cohort study to evaluate the gender income gap in underdeveloped areas in China. We compared the level and change in the income gap between CSP and NCSP graduates and found evidence of a significant gap, with female doctors earning 3.2% (CSP graduates) less and 16.3% (NCSP graduates) less than male doctors in the seventh year after graduation, after adjusting for confounders.

First, gender differences were apparent among young medical school graduates in underdeveloped areas of China, and this difference continuously widened.

Labor market discrimination may be a possible explanation for the observed gender income difference. Gender discrimination still prevails in rural areas. Many elderly individuals continue to believe that male doctors have more extensive experience. This conscious or unconscious gender bias and discrimination lead to lower trust in female doctors, consequently reducing the performance-based income of female physicians. Some studies have also found that gender wage disparities may stem from differences in workloads and work patterns. Theurl and Winner found that male doctors provide more treatments (the average number of treatments per hour) than their female counterparts [10], but our research found no significant statistical difference between male and female doctors in terms of workload. Barry also found that the significant pay disparity between male and female GPs was linked to women spending more time with their patients [33].

Another possible explanation for the gender difference is the “glass ceiling” experienced by women. The concept of a glass ceiling is recurrent in the recent literature on the career problems of women [34–38]. It is defined in Merriam-Webster's Collegiate Dictionary (10th edition) as “an intangible barrier within the hierarchy of a company that prevents women or minorities from obtaining upper-level positions” [39]. A national study of female physicians in academic medicine in the US reported that women were much less likely than men to be promoted; this gender difference persisted even after controlling for work schedule, specialty, and academic productivity, suggesting the existence of a glass ceiling for female physicians [34]. Blumenthal et al. also found that female surgeons were significantly less likely than their male counterparts to be full professors, after adjusting for several factors [40]. Owing to faster career advancement, men earn significantly higher levels of performance-based income than women, resulting in income disparities. However, because of the relatively short observation period, we could not definitively confirm the existence of a glass ceiling effect.

In China, while women have significantly advanced in educational attainment and workforce participation, they continue to face substantial barriers in professional advancement, especially in ascending to senior leadership roles. China boasts one of the highest female labor participation rates globally, with a 71% rate for women over the age of 15 in 2022—far surpassing the global average of 53% [41]. However, research by Qing suggests that women are disproportionately employed in low-skill, low-value positions [42]. Additionally, women typically earn only three-quarters of what men do in similar roles [43]. The "Global Gender Gap Report 2021" shows that China ranks first out of 156 countries in female higher education enrollment rates, yet it lags at 132nd for women in senior roles like legislators, officials, and managers, and even lower at 147th for women ministers—placing in the bottom 20% globally [44]. Notably, there is a lack of research on labor market discrimination and the glass ceiling in the medical field within China, highlighting a critical need for further investigation into gender inequalities in such areas, particularly in developing countries like China.

The proportion of performance-based income was significantly higher for men than women, making it a significant contributor to this disparity. Male doctors often experience the "breadwinner effect" as they bear the financial responsibility of supporting their families. This, in turn, stimulates stronger ambitions and determination in them, fostering a more intense drive to earn money. In contrast, female doctors, influenced by the "caregiver effect" of having children, may be more focused on caring for their children, potentially leading them to overlook performance improvement [9]. Moreover, the remuneration of doctors in China is generally composed of a basic fixed salary, performance-based income, various allowances, and an annual year-end bonus. The level of a doctor's title determines the coefficient for performance and allowances, meaning that higher titles are associated with higher performance-based incentives. Based on research from previously developed countries, it has been found that there is not a significant difference between men and women in terms of fixed income. However, the key variations lie in performance-based income that are linked to factors such as workload and work intensity [10, 11, 17]. Additionally, in China, hospitals primarily derive their revenue from medical services, with only a small portion coming from government financial subsidies. Therefore, to achieve higher earnings, hospitals are more likely to motivate ambitious male doctors by offering them more attractive performance-based income. This, to some extent, elucidates the income disparity observed between male and female doctors. However, this explanation only covers a fraction of the gap, as a more significant factor is likely linked to the previously mentioned gender discrimination.

Second, to some extent, the employment of CSP graduates in PHC roles (PHC institutions with salaried systems) ensures fairness and partially eliminates gender discrimination through targeted training, staffing policies, and accelerated career development paths. After the health system reform in 2009 in China, the salary structure for GPs in PHC primarily consists of a fixed salary [45]. The disparities in fixed salaries are minimal, primarily determined by factors such as educational background, professional title, and years of work experience. Meanwhile, the government offers incentives in the form of policies such as hardship allowances for remote and underdeveloped areas as well as township work subsidies, with relatively minor gender disparities [46]. Additionally, owing to the scarcity of GPs in remote rural areas, there is a limited pool of doctors available for selection [47]. As a result, rural patients cannot choose their contracted GP based on their gender, but they can specify the gender of specialist doctors at the county hospital. This provides equal employment opportunities for female GPs. However, male CSP graduates earn notably less than their NCSP counterparts. This discrepancy makes them more inclined to leave PHC and seek higher-paying professions, driven by pressures from family, society, and other factors [16]. Therefore, male CSP graduates with higher incomes tend to leave PHC. Conversely, those who remain in PHC may experience lower incomes, potentially contributing to a reduction in income disparities between male and female GPs.

In the medical domain, despite female doctors demonstrating excellence and professionalism, they may still encounter gender bias, potentially hindering their opportunities for promotions, well-compensated roles, and other career advancements. Achieving genuine gender equality necessitates not only fostering ambition among male doctors, but also continuous efforts to eradicate various forms of discrimination against female healthcare professionals. Such endeavors play a crucial role in dismantling gender barriers and cultivating a more fair and inclusive working environment within the medical field.

This study had several limitations. First, as the income data were self-reported, we could not verify their authenticity or the presence of any additional income, which may have introduced a reporting bias. Second, as the follow-up surveys were conducted online, the questionnaire was kept relatively concise to ensure completion rates. Few variables were therefore available to decompose the gender income gap. Consequently, we focused exclusively on performance-based income, while other potentially influential factors remain unanalyzed. Third, all cohort studies inevitably encounter some participant loss in follow-up surveys. Our previous research addressed the issue of sample attrition and found that it had a minimal impact on the focal career development issues of the cohort under study [48]. Additionally, considering the significant impact of COVID-19 on healthcare workers in China from 2020 to 2022, our previous research identified an increase in workload among graduates due to the pandemic [49]. However, this study did not measure the impact of COVID-19 on the income of graduates. We will further focus on the impact of COVID-19 on income in our future research.

In conclusion, a gender income gap was apparent in young doctors in underdeveloped areas of China, potentially hindering PHC services from attracting and retaining talent. Compared to NCSP graduates, the income gap was smaller for CSP graduates. The high proportion of performance-based income among men was a main explanation for the observed gender difference. To improve the capacity of rural PHC services and increase health accessibility and equity, a more explicit compensation system must be established to enhance support for female healthcare workers at the system level.

### Supplementary Information

Supplementary Material 1.

## Data Availability

The datasets generated and/or analyzed during the current study are not publicly available due to limitations of ethical approval involving the personal data and anonymity; however, they are available from the corresponding author on reasonable request.

## Abbreviations

CSP: Compulsory services program
NCSP: Non-CSP
GPs: General practitioners
IRB: Institutional review board
CPI: Consumer price index
PHC: Primary health care
SAGER: Sex and gender equity in research

## References

[1] Shannon G, Jansen M, Williams K (2019). Gender equality in science, medicine, and global health: where are we at and why does it matter?. Lancet.

[2] Li M, Raven J, Liu X. Feminization of the health workforce in China: exploring gendered composition from 2002 to 2020. Hum Resour Health 2024;22:15. 10.21203/rs.3.rs-2211899/v1. 10.1186/s12960-024-00898-w PMC10877893 38373975

[3] Woodhams C, Dacre J, Parnerkar I, Sharma M (2021). Pay gaps in medicine and the impact of COVID-19 on doctors’ careers. Lancet Lond Engl.

[4] McKeigue PM, Richards JD, Richards P (1990). Effects of discrimination by sex and race on the early careers of British medical graduates during 1981–7. BMJ.

[5] Ohsfeldt RL, Culler SD (1986). Differences in income between male and female physicians. J Health Econ.

[6] Baker LC (1996). Differences in earnings between male and female physicians. N Engl J Med.

[7] Cheng TC, Scott A, Jeon S-H, Kalb G, Humphreys J, Joyce C (2012). What factors influence the earnings of general practitioners and medical specialists? Evidence from the medicine in Australia: balancing employment and life survey. Health Econ.

[8] Bayati M, Rashidian A, Sarikhani Y, Lohivash S (2019). Income inequality among general practitioners in Iran: a decomposition approach. BMC Health Serv Res.

[9] Mikol F, Franc C (2019). Gender differences in the incomes of self-employed French physicians: the role of family structure. Health Policy.

[10] Theurl E, Winner H (2011). The male-female gap in physician earnings: evidence from a public health insurance system. Health Econ.

[11] Dumontet M, Le Vaillant M, Franc C (2012). What determines the income gap between French male and female GPs—the role of medical practices. BMC Fam Pract.

[12] Hoff T, Lee DR (2021). The gender pay gap in medicine: a systematic review. Health Care Manage Rev.

[13] Wang J, Zhao Q, Liu T, An M, Pan Z (2019). Career orientation and its impact factors of general practitioners in Shanghai, China: a cross-sectional study. BMJ Open.

[14] Ma X, Wang Z, Liu X (2019). Progress on catastrophic health expenditure in China: evidence from China Family Panel Studies (CFPS) 2010 to 2016. Int J Environ Res Public Health.

[15] Meng Q, Mills A, Wang L, Han Q (2019). What can we learn from China’s health system reform?. BMJ.

[16] Zhang B, Wang Z, Hu D, Li M, Wang H, Wei T, Cheng X, Cheng H, Liu X (2023). Longitudinal comparison of order orientation and income of general clinical graduates (Chinese). Chin Gen Med.

[17] Dumontet M, Franc C (2015). Gender differences in French GPs’ activity: the contribution of quantile regressions. Eur J Health Econ HEPAC Health Econ Prev Care.

[18] Ran L, Luo K, Wu Y, Yao L, Feng Y (2013). An analysis of China’s physician salary payment system. J Huazhong Univ Sci Technol Med Sci.

[19] Zhang C, Liu Y (2018). The salary of physicians in Chinese public tertiary hospitals: a national cross-sectional and follow-up study. BMC Health Serv Res.

[20] Hu D, Chen C, Zhang C, Huang M, Wang J, Jia Z, Li H, Liu X (2016). Survey on the implementation of the policy of free training for rural medical students. Chin Health Policy Res.

[21] Li M, Zuo Y, Zhang F, Wang Z, Cheng H, Liu X (2022). Study on the influence of standardized training of resident physicians on the passing rate of the order-oriented medical students' licensed physician qualification examination. Chin Gen Med.

[22] Cheng X, Zhang X, Wang J, Zhang Z, Dou L, Liu X (2022). Employment and compliance of order-directed medical students: Based on a five-year follow-up analysis of four medical schools. Chin Gen Med.

[23] Hatcher AM, Onah M, Kornik S, Peacocke J, Reid S (2014). Placement, support, and retention of health professionals: national, cross-sectional findings from medical and dental community service officers in South Africa. Hum Resour Health.

[24] Mansoor GF, Hashemy P, Gohar F, Wood ME, Ayoubi SF, Todd CS (2013). Midwifery retention and coverage and impact on service utilisation in Afghanistan. Midwifery.

[25] Joarder T, Rawal LB, Ahmed SM, Uddin A, Evans TG (2018). Retaining doctors in rural Bangladesh: a policy analysis. Int J Health Policy Manag.

[26] Whaley CM, Arnold DR, Gross N, Jena AB (2020). Practice composition and sex differences in physician income: observational study. BMJ.

[27] Weeks WB, Paraponaris A, Ventelou B (2013). Sex-based differences in income and response to proposed financial incentives among general practitioners in France. Health Policy.

[28] Yang J (2020). Women in China moving forward: progress, challenges and reflections. Soc Incl.

[29] Women and socioeconomic status. https://www.apa.org. https://www.apa.org/pi/ses/resources/publications/women . Accessed 30 Aug 2023.

[30] Qing S (2020). Gender role attitudes and male-female income differences in China. J Chin Sociol.

[31] Weaver AC, Wetterneck TB, Whelan CT, Hinami K (2015). A matter of priorities? Exploring the persistent gender pay gap in hospital medicine. J Hosp Med.

[32] French F, Andrew J, Awramenko M (2006). Why do work patterns differ between men and women GPs?. J Health Organ Manag.

[33] Barry J (2021). Real wage growth in the US health workforce and the narrowing of the gender pay gap. Hum Resour Health.

[34] Tesch BJ, Wood HM, Helwig AL, Nattinger AB (1995). Promotion of women physicians in academic medicine: glass ceiling or sticky floor?. JAMA.

[35] Gómez-Durán E, Gassó AM, Bisbe E, Virumbrales M (2023). Women in Spanish institutional medicine leadership: the glass ceiling remains seemingly invulnerable. Med Clin (Barc).

[36] Achkar E (2008). Will women ever break the glass ceiling in medicine?. Am J Gastroenterol.

[37] Fernández-Guerrero IM (2022). Researching while being a woman in emergency medicine: where is the glass ceiling?. Emerg Rev Soc Espanola Med Emerg.

[38] Fader AN, Wang KC, Wethington SL (2022). The glass ceiling in obstetrics and gynecology: breakable but still a barrier. J Minim Invasive Gynecol.

[39] McManus IC, Sproston KA (2000). Women in hospital medicine in the United Kingdom: glass ceiling, preference, prejudice or cohort effect?. J Epidemiol Community Health.

[40] Blumenthal DM, Bergmark RW, Raol N, Bohnen JD, Eloy JA, Gray ST (2018). Sex differences in faculty rank among academic surgeons in the United States in 2014. Ann Surg.

[41] World Bank Open Data. World Bank Open Data. https://data.worldbank.org. Accessed 15 May 15 2024.

[42] Qing S. Gender discrimination in job promotion (Chinese). Manag World. 2011;28–38. https://kns.cnki.net/kcms2/article/abstract?v=vAdbs87d_Ck4rNxcaquPps8m_Oan2gxUwI_2BZfFpKqa6y8yvZYhZk3aj26-EatEEYbSxChPQwjqaYp2DOneREqaaMXu-PIfaqXvXxBL1gHQWk45iFFTleIt9DAx5y0PPrDW1Z2cznU=&uniplatform=NZKPT&language=CHS

[43] Peng X, Zou X, Fu Y. Research progress of human capital theory from the perspective of gender difference (Chinese). Econ Dyn. 2024;145–60. https://kns.cnki.net/kcms2/article/abstract?v=vAdbs87d_CkCKhmBvB7cuAlPW6KZhJxFBRf8HDmYtl_NqQYkJK9PnVYmHOddNSdOQcjW7oAlJm12pMT6oKJtu7QnUNSALRAV4VYQeyg1vgbzUoABGid3EN3Vg2JXcxTHKdevgPtBboify8RECkP0w==&uniplatform=NZKPT&language=CHS

[44] World Economic Forum. Global Gender Gap Report 2021. World Econ. Forum. 2021. https://www.weforum.org/publications/global-gender-gap-report-2021/in-full/. Accessed 15 May 2024.

[45] Hu L, Cao Y, Wang B, Liu Y, Rao K (2015). Discussion on the reform of health service payment system in primary medical institutions in China. Chin J Hosp Admin.

[46] National Health Commission of the People's Republic of China. Notice on job placement and performance management of rural order-oriented free training medical students (Chinese). Departmental documents of The State Council. https://www.gov.cn/zhengce/zhengceku/2019-11/13/content_5451684.htm. Accessed 10 Sep 2023.

[47] Li X, Cochran C, Lu J (2015). Understanding the shortage of village doctors in China and solutions under the policy of basic public health service equalization: evidence from Changzhou. Int J Health Plann Manage.

[48] Li M, Wang Z, Zhang B, Wei T, Hu D, Liu X (2022). Sample attrition analysis in a prospective cohort study of medical graduates in China. BMC Med Res Methodol.

[49] Cheng H, Wang Z, Zhang B (2021). Rural medical graduates participated in the research on the prevention and control of the novel coronavirus pneumonia epidemic. Chin Gen Med.

