# Supplementary material for: Gender income differences among general practitioners with compulsory services in early career stage in underdeveloped areas: evidence from a prospective cohort study in China
Source: Hum Resour Health. 2024 Jun 27;22:45. doi: 10.1186/s12960-024-00930-z (PMC11209954; doi:10.1186/s12960-024-00930-z)
Supplement: Supplementary file 1 — Supplementary Material 1. [file 12960_2024_930_MOESM1_ESM.docx]

**Gender income differences among general practitioners with compulsory services in early career stage in underdeveloped areas: evidence from a prospective cohort study in China**

*Supplementary file*

**Table S1** The socioeconomic background of the three provinces, CNY

|  | Qinghai | Jiangxi | Guangxi | National |
| --- | --- | --- | --- | --- |
| GDP 2022 (¥100 million) | 3610 | 32075 | 26301 | 1210207 |
| Per capita GDP | 60724 | 70923 | 52164 | 85698 |
| Household consumption level | 22389 | 21482 | 19959 | 28537 |

Notes: Numbers adjusted for inflation and measured in Chinese yuan in 2022 prices
